# Artificial Diet for Immatures of *Sphenophorus levis* Vaurie, 1978 (Coleoptera: Curculionidae), Based on a Multidimensional Model

**DOI:** 10.3390/insects15120944

**Published:** 2024-11-29

**Authors:** Luana Viana Faria, Tamara Akemi Takahashi, Adriano Gomes Garcia, José Roberto Postali Parra

**Affiliations:** Department of Entomology and Acarology, “Luiz de Queiroz” College of Agriculture, University of São Paulo (USP), 11 Av. Pádua Dias, Piracicaba 13418-900, São Paulo, Brazil; tamaratakahashi@gmail.com (T.A.T.); adrianogomesgarcia@gmail.com (A.G.G.);

**Keywords:** sugarcane weevil, Design-Expert^®^, fecundity, sugarcane

## Abstract

Pest attacks, especially by *Sphenophorus levis*, are a factor affecting sugarcane production. As the pest’s life habits make chemical control difficult, efforts are focused on finding integrated management alternatives. It is therefore essential to study the biology of *S. levis*, using artificial diets for rearing the insect in the laboratory. Artificial diets are commonly used for rearing pest insects, providing specimens year-round even when they are present only seasonally in the field, as is the case with *S. levis*. However, there is no diet in the literature that provides viable egg-to-adult development for rearing pests in the laboratory. Recently, studies to optimize diets using software with an interactive approach have shown good results in diet development. This study aimed to develop an artificial diet for *S. levis* using Design-Expert^®^ software (Version 11) to enable development and viability comparable to insects in the wild. The multivariate approach consisted of initially varying six components of the diet and then varying the three components that most influenced viability and the development rate, providing viabilities of over 60% at the end of the *S. levis* cycle in both phases of the screening design. However, the physical characteristics of the diet, such as the water content and texture, proved to be preponderant factors for the proper development of the insect and should be considered when using its artificial diet using a suitable container and uncrushed wheat germ.

## Simple Summary

Despite the high productivity of the sugarcane crop, attacks by pests is one of the main factors affecting crop yields. Among them, the curculionid Sphenophorus levis has become very important, with management made difficult by the pest’s habit of life. The study of insect biology is essential for year-round research in order to provide more effective control. In the literature, only one artificial diet is available for *S. levis*, with viability only for the larval stage, making it difficult to study the pest. This research therefore used response surface models, using Design-Expert^®^ software (Version 11), to optimize a new artificial diet that would provide viable adults capable of reproducing in the laboratory. Using the program, it was possible to identify diet components that acted positively and negatively on the viability and larval development variables by varying six components of the initial diet studied (casein, agar, wheat germ, sugar cane bagasse, sucrose and nipagin). The casein and agar components acted positively on the development of *S. levis*, while the sugarcane bagasse component acted deleteriously. It was possible to find egg-adult viability of over 60% in the two diet combinations tested by the Software.

## 1. Introduction

The genus *Sphenophorus* belongs to the Curculionidae family and comprises a complex of species that occur on several continents, especially in North America because they cause economic damage to certain species of grass, especially some varieties of commercial grasses [1,2,3]. In Brazil, the sugarcane weevil *Sphenophorus levis* Vaurie, 1978 (Coleoptera: Curculionidae), is one of the main pests affecting the sugarcane crop in the country’s largest producer, the state of São Paulo (SP), all of them causing huge losses without specific percentage assessments [4,5,6].

The importance of this pest was first recognized in 1977, when an outbreak occurred in Santa Bárbara d’Oeste, SP, damaging the local sugarcane crop and killing 50–60% of the tillers that were still in the cane-plant stage. The weevil was erroneously described as a new species in 1978 [7] and has now spread to other states, including São Paulo, Santa Catarina [8], Minas Gerais [9], Mato Grosso do Sul [10], Paraná [11], Mato Grosso, and Goiás [12], in all causing major losses without specific percentage assessments.

The first artificial diet for the pest was developed by Degaspari and coworkers [13], derived from the diet described by Singh [13] for *Sphenophorus venatus vestitus* Chittenden (1904). However, this formulation did not adequately support the development from egg to adult, resulting in slow development and low adult fecundity.

To control *S. levis* more effectively in the field, knowledge of the insect’s biology is essential [8,14]. Laboratory rearing is fundamental for research and the development of pest detection and control methods [15,16]. Despite the importance of this pest, little is known about its biology due to the difficulty of rearing it in the laboratory for successive generations. In this sense, it is essential to develop an efficient rearing method that includes an artificial diet that will allow continued studies of this pest, which includes testing with biological control agents [14]. To date, there are no viable artificial diets in the literature for rearing *S. levis* in the laboratory in Brazil.

Studies with different software programs to optimize artificial diets for different species using an interactive approach have yielded good results for biology studies [17,18]. To develop an artificial diet, conventional approaches involve empirical one-factor-at-a-time experiments and multivariate experiments based on mixture designs [19]. The multivariate geometric approach, in combination with a diet mixture design, allows the simultaneous variation in several diet components and reveals important interactive effects of diet components on several responses measured at the same time [20,21], optimizing the response time of the variables studied for an adequate diet.

In this study, by analyzing the performance of three artificial diets used to maintain the larval stage of *S. levis*, it was possible to identify the main key ingredients that provided the best response in relation to the development from egg to adult of the insect using the diet from Ref. [13], compared to another diet with few changes in the amounts of proteins, carbohydrates, and methylparahydroxybenzoate with the exclusion of formaldehyde used in the academy and the most promising diet according to this study.

Based on this information, six components were selected and used to optimize the diet combination, using Design-Expert^®^ (Stat-Ease, Inc., Minneapolis, MN, USA) software, combining the response urface method with a mixture design to identify the main drivers of larval development, maximizing the larval survival and the development rate. The aim was to maintain a population of *S. levis* in the laboratory with a diet that provided high viability and produced insects with reproductive capacities capable of copulating and producing viable eggs under laboratory conditions.

## 2. Material and Methods

### 2.1. Obtaining and Maintaining Sphenophorus levis Vaurie

The *Sphenophorus levis* eggs used to start the laboratory colony were initially supplied by the Sugarcane Technology Center (CTC, Piracicaba, Brazil) from *S. levis* adults collected in the field in March, April, and May 2020 and November, December, January, and February 2021 and 2022 using baits of sugarcane stalks cut in half (30 cm), placed at the base of the clump, and covered with sugarcane straw to capture the adults after the stalks remained in the field for seven days. The adults were collected in sugarcane fields in the regions of Piracicaba, Iracemapolis, Jaboticabal, and Ribeirão Preto in the state of São Paulo in 2020, 2021, and 2022.

The insects collected in the field were separated by sex and placed in cages made from 2.5 L plastic boxes, with approximately 25 couples per cage. Each cage received a stalk of raw sugar cane approximately 15 cm long and cut in half lengthwise. The eggs were collected from the sugar cane with a serrated knife and a fine bristle brush dipped in a 1% copper solution to avoid contamination and deposited in 6 cm Petri dishes containing filter paper moistened with distilled water, with approximately 30 eggs per dish. The surface was sterilized with a 1% solution of methyl parahydroxybenzoate (nipagin) also to avoid contamination. The plates were transferred to an incubator set at 25 °C, RH 60 ± 10%, and 14 h photophase until the larvae hatched.

All the larvae used in the experiments in this study belonged to the first generation after the field collection.

### 2.2. General Structure of the Experimental Project

An initial pilot test was conducted to compare three diets obtained from different sources that have been used to maintain larvae of *S. levis* in the laboratory. These were Diet A, used in the Insect Biology Laboratory (Luiz de Queiroz College of Agriculture, Piracicaba, São Paulo, Brazil); Diet B, used in industrial mass rearing; Diet C, taken from the literature [13]. The aim was to select the most promising diet to continue the experiment. Each larva aged < 24 h was counted as a repetition and placed in a separate flat-bottomed glass tube containing one of the diets and, the tube was capped with water-repellent absorbent cotton. Each diet tested had 40 repetitions, and a total of 40 individualized larvae were used. The tubes were placed in air-conditioned chambers at 25 ± 2 °C, RH 60 ± 10%, and 14 h photophase until the the larvae completed development.

Two parameters were calculated: the insect survival and development rate, given by 1/(development time), covering all larval stage and pupal stages. All data were tested for homogeneity [22], normality, and independence of residuals [23]. Insect survival was analyzed using generalized linear models (GLMs) with binomial distribution. Given its non-parametric nature, the development time was analyzed using the Kruskal–Wallis test, followed by Dunn’s test for pairwise comparisons.

After the most promising diet was selected according to the insects’ survival, an interactive approach was used to develop an artificial diet by screening six components of the initial diet (unground wheat germ, casein, sucrose, sugarcane fiber, Nipagin, and agar). (Using ground wheat may affect the consistency of the diet.) The nutritional profiles of the *S. levis* diet mixtures were explored by varying these primary components in addition to an anti-contaminant, using Design-Expert® software (Stat-Ease, Inc., Minneapolis, MN, USA). 

Linear equations were generated to describe the effect of each diet component on the responses to be studied (larval + pupal survival and development rate). In the selection design carried out by the software, the aim was to identify the components that most affected these responses. Subsequently, a mixture model of different quantities of these components in the diets was used to identify an optimal artificial formulation with the main components that most affected the responses. The other components of the diet were kept fixed based on the diet that provided the highest survival and development rate, according to the results of the first diet selection.

### 2.3. Exploratory Selection of Three Artificial Diets, Based on a Primary Diet [14]

We used three diets: (A) supplied by the Insect Biology Laboratory of ESALQ-USP, Piracicaba; (B) used by the industry; (C) found in the literature [13], which have been used to keep insects alive at least during the larval stage. These diets consisted of a mixture of solids (wheat germ, casein, sucrose, sugarcane fiber, Wesson salts, and agar), liquids (vitamin solution (niacinamide, calcium pantothenate, riboflavin, biotin, thiamine HCI, pyridoxine HCI, folic ccid and vitamin B12), potassium hydroxide (KOH) solution, and water), and anti-contaminants (Nipagin, ascorbic acid, and sorbic acid). The vitamin mixture was prepared by mixing the wet route with the liquid route, dissolving the vitamin content of the dry route in 1 L of distilled water, and then adding the content of the wet route, which was homogenizing, and storing in a refrigerator. The potassium hydroxide (KOH) solution was prepared by dissolving 11 g of potassium hydroxide in 200 mL of distilled water and was stored at room temperature.

### 2.4. Selected Diet Plus Six Components Indicated by Design-Expert^®^ Software

Originally, the diet consisted of 13 ingredients, but based on the results of the exploratory experiment, the six components that varied the most in other artificial diets for lepidopterans and curculionids [15,24] were chosen. The other ingredients (vitamins, salt mixtures, and antibiotics) were kept constant at the same levels as in the selected diet (Diet B).

Then, using the best diet from the first selection, which was renamed SL-1.0, to optimize a multicomponent mixture, a multivariate geometric design combined with response–surface modeling was used in a new experiment, varying only these six components of the artificial diet: casein, sugarcane fiber, sucrose, wheat germ, Nipagin, and agar. The exploratory design was created with Design-Expert software resulting in 16 design tests divided into four blocks; each block has a central point, also used as a reference point by the Software, in which they are arranged geographically as comparison parameters for the other diets tested. Replicating the centroid contributes to the robustness of the experimental design by confirming that the central point is stable and reliable across multiple runsEach test was started with 80 newly hatched larvae (<24 h old) in individual flat-bottomed glass test tubes plugged with absorbent cotton. The survival and development rates (1/development time) of the larvae and pupae were observed until the adults emerged. The diet that resulted in the highest viability and development rate (larval + pupal stages) at this stage was labeled SL-2.0 (Table 1).

### 2.5. Design of a Three-Component Mixture for the Artificial Diet

The three components responsible for the largest effects on immature survival and development rate (larvae + pupa) in the six-component screening step were used to construct a new combination using Scheffé’s special cubic response–surface models. Given the inherent complexity of diet mixtures, it was necessary to employ a multivariate optimization technique designed specifically to facilitate navigation in n-dimensional response spaces. This experimental design consisted of 20 points divided into four blocks (Table 1). Other ingredients were kept constant, following the composition of the SL-2.0 diet. The optimal composition at this stage (SL-3.0) was predicted using the contour plots produced by the Design-Expert software (Table 2).

### 2.6. Test of Water Loss from the Artificial Diet

In an attempt to reduce the loss of water from the medium observed in the diet optimization experiments, which caused the immature stages to dry out and die, a new experiment was carried out with three different kinds of containers using the SL-3.0 diet determined from the diet optimization experiment using the Design-Expert program. The aim was to determine the loss of water from the diet in the different containers, as well as possible loss with different materials used to seal the containers. These were a flat-bottomed glass tube (2.3 cm in diameter × 8 cm high) plugged with absorbent cotton; a flat-bottomed glass tube (2.3 cm in diameter × 8 cm high) sealed with plastic film; a 50 mL Falcon tube closed with the original cap. The diet was prepared in the same way as in the previous experiments and poured into each container. The initial weighing was conducted with the fresh diet, and water loss was assessed by weighing each container on a digital scale daily for 40 days.

### 2.7. Comparison of the Selected Diets

To validate the results, all the diets produced (SL-1.0, 2.0, and 3.0) were evaluated simultaneously. Each diet was tested with four replicates; each started with 20 newly hatched larvae in individual tubes sealed with plastic film. The following parameters were calculated: insect survival and development rate, which is given by 1/(development time), covering all larval and pupal stages. All data were tested for homogeneity [22], normality, and independence of residuals [23]. The data were analyzed using generalized linear models (GLMs) with a Poisson distribution for the mean development time. Survival data were analyzed using GLM with binomial distribution.

All the diets were prepared in the same way as the previous ones and transferred to flat-bottomed test tubes. In this case, the tubes were sealed with plastic film since preliminary tests indicated that this type of sealant maintained higher water contents. Adequate water content is a major factor for increasing viability in the pre-pupa stage, even though the diet loses large amounts of water due to the insect’s long development time.

## 3. Results and Discussion

### 3.1. Exploratory Selection of Three Artificial Diets, Based on Primary Diet [13]

The results for diet B (Item 2.3) showed that the insects completed development in 77.2 ± 2.3 days; however, it was with low viability of 32.5 ± 6.6%. Diets A and C, on the other hand, resulted in a high percentage of mortality in the first instars (Diet C) or high mortality due to fungal contamination and mite infestation during larval development (Diet A).

**Figure 1:**
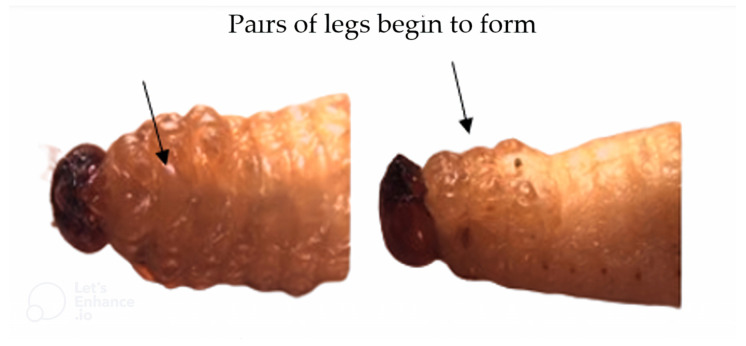
*Sphenophorus levis* larvae that died naturally in the prepupa stage. Temperature 25 ± 2 °C, RH 60 ± 10%, photophase 14 h.

The high mortality could be assessed visually in the pre-pupal stage when the larva stopped feeding and the three pairs of legs began to form; most of the larvae already had an open pupal chamber (Figure 1).

Despite the importance of *S. levis* as a key pest in sugarcane cultivation, few studies have elucidated its biology and behavior [25,26]. Since the publication of Degaspari and collaborators [13], no study on the development of an artificial diet that provides acceptable viability and fertile adults has been published. The artificial diets currently used provide acceptable viability for the development of larvae but do not produce an acceptable number of adults from the diet that provide fertile eggs [13].

During the period following the publication of the first artificial diet for *S. levis*, there was no research recorded in the literature examining the artificial diet, questioning the basic assumptions about the quality of insects reared in colonies or the applicability of the results obtained with the artificially reared sugarcane weevil.

The little information on the development and behavior of the insect consists of preliminary observations, mainly on insects from field conditions, without knowing the age of the insects and using methods that are difficult to interpret [8,26]. Other diets formulated according to Singh [15] used in the Insect Biology Laboratory and in industry are well accepted by *S. levis* in the larval stage but result in high mortality in the final stage of the cycle. This poor performance may be related to a nutritional imbalance.

### 3.2. Selected Diet Plus Six Components Indicated by Design-Expert Software

Diet B, which provided the highest viability at the end of the cycle, even if it was below 40%, was chosen to continue with the next phases, in which six components of the diet were varied simultaneously: agar, sucrose, wheat germ, casein, sugarcane bagasse, and Nipagin. The initial diet contained carrageenan in its composition, but after observing characteristics such as consistency and pupal chamber formation, agar was used as the standard component for all subsequent diets. Pure agar produced better consistency over time and less water loss.

**Figure 2:**
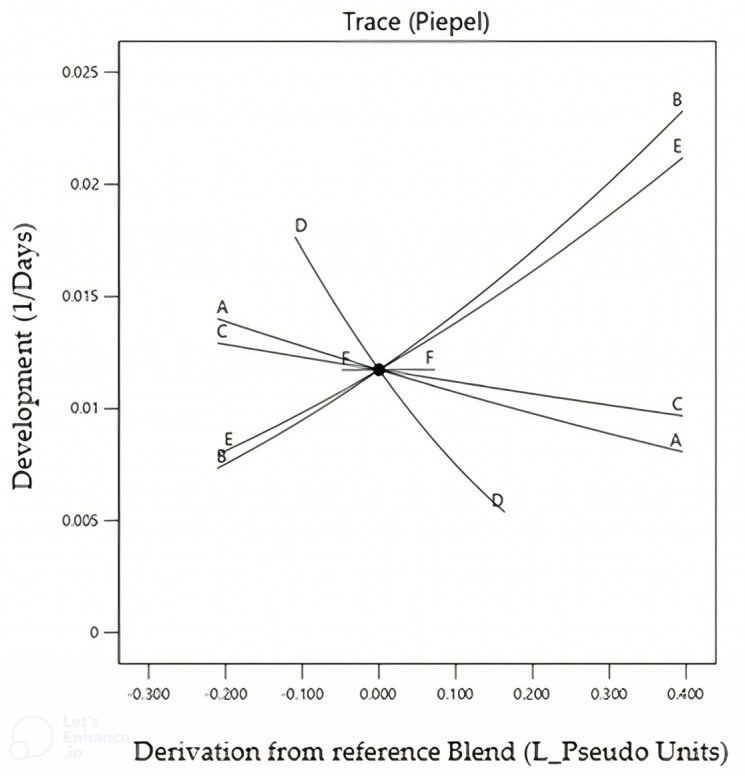
Development of *Sphenophorus levis* on sixteen artificial diet combinations, varying the components: A—wheat germ; B—casein; C—sucrose; D—sugarcane fiber; E—agar; and F—methyl parahydroxybenzoate (Nipagin^®^). Temperature 25 ± 2 °C, RH 60 ± 10%, photophase 14 h.

This was due to the difference in the carrageenan and agar molecules; according to Norziah and coworkers [27], the agar molecule retains more water than the carrageenan molecule. The six-component selection design using the Design-Expert program produced significant linear response models for development time (*p* ≤ 0.05, F_5.11 = 3.84) with R^2^ = 0.73, but not for survival (*p* = 0.8849, F_5.11 = 0.32), indicating that the relationship between this response and the component is not linear (Figure 2).

### 3.3. Design of a Three-Component Mixture for the Artificial Diet Based on 3.2

Based on the analysis of the Cox graph (Figure 2), three components with the highest expression were selected by the Design-Expert program for the next mixture designs, based only on the response model for development time. The models indicated that casein, agar, and sugarcane fiber had the largest effects on improving these criteria.

This relationship between each component and the development time shows that the slope is directly proportional to the magnitude of the effect of the individual factors on the measured response variable. It was apparent that casein and agar were positive factors, i.e., increasing these components led to faster development, while sugarcane fiber slowed the rate of development, even if the concentration was close to the upper limit tested in the best diet. Based on the 16 diets tested in the selection experiment, the formulation at the center point provided the shortest development time, and then different concentrations for the three selected components were tested.

The Cox effect observed in the screening study showed that varying the proportions of the six initial ingredients affected the diet performance for multiple responses compared to the reference diet. The main result was a simplified diet with greater viability compared to the previous diet. However, some ingredients showed detrimental effects, while others, although beneficial, were present in higher proportions than ideal for insect development. Thus, the multidimensional approach helped to avoid confusion caused by simultaneous changes in ingredients and made it possible to study the interactions among them over the entire space of the application between these ingredients [28]

The design of the diet mix using the Design-Expert program produced significant response surface models for survival (*p* < 0.05, F 3.20 = 5.34) in the optimization phase. However, there were no significant effects on the development rate (*p* = 0.09, F 3.20 = 3.09) when varying the three different components of the best artificial diet generated in the previous phase, varying casein, agar, and sugar cane fiber.

The nutritional content of artificial diets can reduce development time and increase the viability of insects, which is in agreement with results from other studies that evaluated other pests [29,30]. In the present study, the development time was similar to that found by other authors studying *S. levis* [8,13].

**Figure 3:**
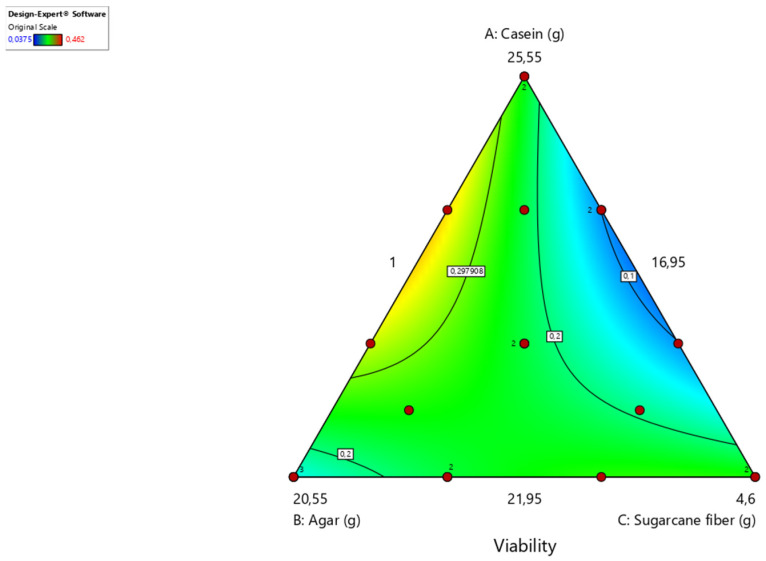
Contour graph indicating different combinations of the three components evaluated: casein (A), agar (B), and sugarcane fiber (C). The lighter yellow regions indicate combinations that resulted in higher viability. Composed using the Design-Expert program (Stat-Ease, Inc., Minneapolis, MN, USA). Temperature 25 ± 2 °C, RH 60%, photophase 14 h.

However, it is not yet clear whether the development time in the laboratory is related to the development time in the field. The relationships between ingredients and survival are shown in contour plots, in which the magnitude of the response variables is coded in different colors. Values close to yellow give greater viability to the insect’s development, which is inversely proportional to values close to blue (Figure 3).

As inferred from the contour graph, high proportions of sugarcane fiber reduced larval survival, which is why this parameter was set at the minimum value tested by the program (1.0 g). Intermediate values of casein and agar within the range tested implied an increased viability of larvae. However, the other experiments showed that sugarcane fiber, although it has a deleterious effect on this weevil, is essential for phagostimulant action and in the physical factors of the diet, helping to form the pupal chamber in the last stage of the insect.

The application of geometric and mathematical approaches, such as response surface modeling and its combination with n-dimensional mixture designs, has also been used to improve existing diets for other insect species, optimizing the response time of the variables used, as in this research, especially in long-cycle insects such as coleopterans, in agreement with other studies [17,20,31,32]. According to Himuro and coworkers [33], artificial diets have significant physiological and morphological effects that result in adequate reproductive characteristics in the adult stage.

### 3.4. Artificial Diet Water Loss Test According to the Results in 3.3

Due to the insect’s long period of development, the diets lost large amounts of water and dried out at crucial stages of development, especially in the pre-pupal stage. As a result, mortality increased in the pre-pupal and pupal stages.

**Figure 4:**
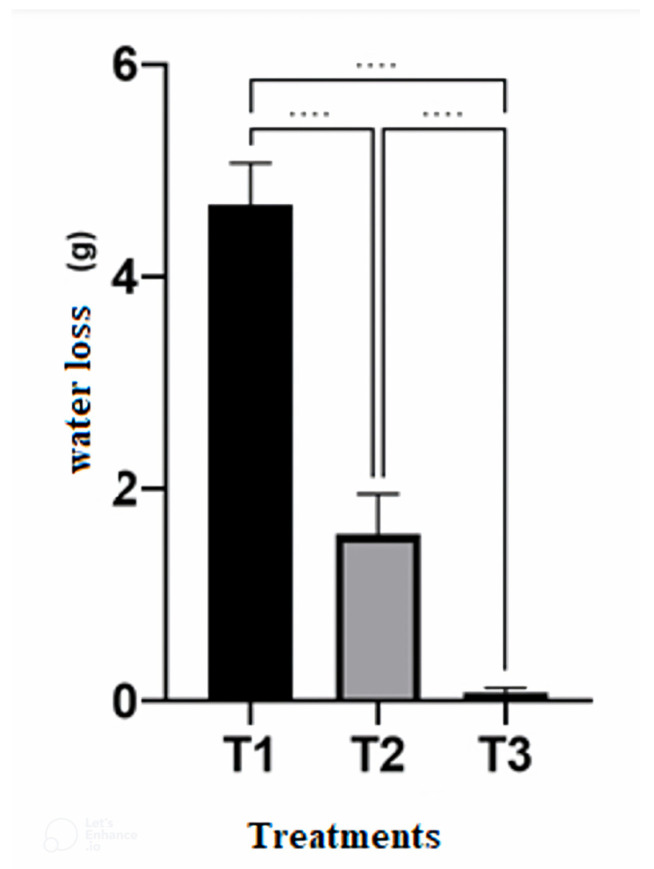
Mean and standard deviation of water loss (g) from the artificial diet in the different containers. T1: Glass tube (plugged with absorbent cotton); T2: Glass tube (sealed with plastic film); and T3: Falcon tube with the original cap. Temperature 25 ± 2 °C, RH 60 ± 10%, photophase 14 h. **** indicates *p* < 0.0001 by One-Way ANOVA and Tukey’s post-test.

With respect to water loss from the diet, the treatments differed from each other. The Falcon tube or similar container lost the least amount of water, followed by the glass tube sealed with plastic wrap and the glass tube plugged with absorbent cotton, respectively (Figure 4).

In additional tests, the insects did not complete development in the Falcon tube closed with the original lid, with 100% mortality and no consumption of the diet. Therefore, the glass tube sealed with plastic film was chosen to continue all stages, as this arrangement performed best for the development of *S. levis* and resulted in a lower contamination rate compared to the glass tube capped with absorbent cotton. These tests proved that adequate water content is essential in the pre-pupation period when *S. levis* stops feeding and opens a gallery in the diet.

In addition to the nutritional balance of the diet, maintaining the water content of the diet was fundamental for increasing the viability of the artificial diet. Using the most suitable container (glass tube sealed with plastic film (PVC film)) ensured that the water content of the artificial diet was maintained until the insect emerged. Other authors have shown that, in curculionids, water content significantly affects the increase in mass and length of individual larvae after three weeks [34] and is, therefore, an important physical factor in the development of the pest.

### 3.5. Comparison of the Selected Diets According to the Software Design Expert

When the three diets tested in all phases of the study were compared to each other using the best container for delaying water loss (glass tube sealed with plastic film), significant differences were observed for both survival (F_2.8 = 31.847, *p* < 0.01) and development rate (F_2.8 = 6.951, *p* < 0.05) of SL-2 and SL-3 compared to SL-1. However, even though the average values observed for the SL-3.0 diet were the most promising, there was no significant difference between SL-2.0 and SL-3.0 (Table 3) in the variables studied.

From the joint analysis of the diets tested, together with observations of the physical characteristics that affect the development of the larvae and pupae, such as water content and texture of the diet for the formation of the pupal chamber of the essential insect in this species, based on the non-significance between (SL-2.0 and SL-3.0) we can infer that a composition with values of casein, agar and sugar cane fiber in the range of 21.95 g to 24.35 g, 16.95 g and 18.15 g and 1 g and 4.6 g respectively are acceptable based on the diets that provided the greatest survival (SL-2 and SL-3).

However, as shown by the software used, high values of sugarcane fiber can be harmful to the development of the insect, and, therefore, intermediate values within the indicated range are recommended. The other components remained constant, based on the diet that provided the greatest viability in the screening experiment (Table 4).

The time spent formulating and developing insect diets based on empirical and multivariate experiments based on mixture designs, in which each component of the diet is varied independently [19], is not very useful for developing artificial diets for long-cycle insects. In this context, the use of response surface methodology combined with mixture design for artificial diets led to a good result for the development of *S. levis*, as well as for other coleopteran species that have a long development cycle [17,20].

Lapointe and collaborators [31], who varied between three ingredients in the artificial diet of the root weevil *Diaprepes abbreviatus* (L.) (Coleoptera: Curculionidae) and also used response surface methods based on multidimensional models to optimize the rearing of the pest in the laboratory and increase the fecundity of the insects, obtained good results in increasing the viability of the insects in the same way as in the present study in which the diets SL-2.0 and SL-3.0 diets achieved viability above 60% and did not differ significantly, providing reproductive adults that had the ability to copulate with each other and that produced viable eggs in the substrate used for oviposition (raw sugar cane) using the Design Expert software.

The multidimensional analysis found a nutritional balance in the diet of *S. levis*, mainly between the proportions of carbohydrates and proteins, which are related to the growth of the larvae through proteins, as well as the energy demanded by the insect through the regulation of carbohydrates. Other authors studying mathematical modeling for nutritional balance in social insects [35,36] have shown that a lack of balance between dietary ingredients can deregulate population dynamics and the allocation of foraging tasks in social insect colonies and Longevity.

In this research, it was possible to optimize the response time of the variables studied together, obtaining a diet that allowed the rearing of the first laboratory generation. Providing a subsidy for future research with the pest in laboratory conditions. To our knowledge, this is the first study using this method to model and maximize response time in the development of an artificial diet for *S. levis* using the multivariate approach. However, this approach has been used in several studies to optimize other biological activities [37,38].

## 4. Conclusions

The program Design-Expert™ provided a new approach in the search to optimize an artificial diet for rearing *S. levis*. After the trials, both the SL-2 and SL-3 diets provided viability above 60% and can be used for rearing the insect in the laboratory. However, physical characteristics, such as an appropriate container for keeping water content during the critical stages of immature development, have proved to be essential. Although sugar cane fiber has a phagostimulating effect and is essential for the formation of the pupal chamber by the insect, it is necessary to be cautious when adding this compound close to the maximum amount considered (4.6 g) due to its deleterious effects observed in this study.

The diets are sustainable for rearing *S. levis* throughout generations, which could allow more studies regarding the biology of the species and how to manage them in the field since now it is possible to standardize the bioassays and then assess the effect of control methods.

## Figures and Tables

**Table 1:** Proportions of six diet ingredients varied in a component-mixture experiment (casein, sugarcane fiber, and agar) to assess the development of *Sphenophorus levis*. based on the Design-Expert^®^ program (Stat-Ease, Inc., Minneapolis, MN, USA).

Wheat Germ (g)	Casein (g)	Sugar (g)	Sugarcane Fiber (g)	Agar (g)	Nipagin^®^ (g)
21.95	21.95	16.95	4.6	16.95	2.6
15	35	10	1	19	5
15	15	30	10	10	5
35	19	10	10	10	1
15	15	19	1	30	5
21.95	21.95	16.95	4.6	16.95	2.6
28	35	10	1	10	1
15	19	30	10	10	1
21.95	21.95	16.95	4.6	16.95	2.6
15	25	10	10	10	5
28	15	10	1	30	1
35	15	19	1	10	5
15	28	30	1	10	1
15	19	10	10	30	1
35	15	10	10	10	5
21.95	21.95	16.95	4.6	16.95	2.6

Components with fixed quantities in the diet: Wesson salts 7.5 g; Ascorbic acid 3 g; Sorbic acid 1.17 g; Tetracycline 0.128 g; vitamin solution 7.5 mL; KOH solution 3.67 mL; water 825 mL.

**Table 2:** Proportions of three diet ingredients varied in a three-component mixture experiment (casein, sugarcane fiber, and agar) to assess the development of *Sphenophorus levis*. Analyzed using the Design-Expert^®^ program (Stat-Ease, Inc., Minneapolis, MN, USA).

Casein (g)	Sugarcane Fiber (g)	Agar (g)
21.95	3.4	18.15
21.95	1	20.55
21.95	1	20.55
23.15	3.4	16.95
24.35	1.6	17.55
21.95	1	20.55
23.15	1	19.35
25.55	1	16.95
22.55	3.4	17.55
24.35	1	18.15
23.15	2.2	18.15
21.95	4.6	16.95
21.95	2.2	19.35
21.95	2.2	19.35
23.15	2.2	18.15
22.55	1.6	19.35
25.55	1	16.95
21.95	4.6	16.95
24.35	2.2	16.95
24.35	2.2	16.95

Components with fixed quantities in the diet: Wesson salts 7.5 g; Ascorbic acid 3 g; Sorbic acid 1.17 g; Tetracycline 0.128 g; vitamin solution 7.5 mL; KOH solution 3.67 mL; water 825 mL.

**Table 3:** Survival (%) and total development time observed for the larval + pupal period of *Sphenophorus levis* on the different diets developed during this study. Temperature 25 ± 2 °C, RH 60 ± 10%, photophase 14 h.

Diet	Survivorship (%)	Development Time (Days)
SL-1.0	20.0 ± 4.6 a	70.2 ± 1.94 a *
SL-2.0	62.5 ± 5.95 b	62.4 ± 1.85 b
SL-3.0	67.5 ± 2.89 b	61.9 ± 1.22 b

* Averages followed by different letters differ by Tukey’s test (*p* < 0.05).

**Table 4:** Components of the optimum artificial diet for *Sphenophorus levis*, based on multidimensional models using the Design-Expert program. Temperature 25 ± 2 °C, RH 60%, photophase 14 h.

Components	Quantity
Casein	21.95–24.35 g
Wesson salts	7.5 g
Sugar	16.95 g
Wheat germ	21.95 g
Sugarcane fiber	1–4.6 g
Metilparahidroxibenzoate (Nipagin)	2.6 g
Ascorbic acid	3.4 g
Sorbic acid	1.17 g
Tetracycline	0.128 g
Vitamine solution ^1^	7.5 mL
Agar	16.95–18.15 g
Potassium hydroxide solution (KOH) ^2^	3.67 mL
Total water	525 mL

^1^ Vitamin solution: 1 g niacinamide + 1 g calcium pantothenate + 0.50 g riboflavin + 0.25 g thiamine + 0.25 g pyridoxine + 0.10 g folic acid + 0.02 mg biotin + 2 mL Vitamin B12 (1000 mg/mL). ^2^ KOH solution: 2.25 g KOH + 10 mL distilled water.

## Data Availability

The data sets generated and/or analyzed in the current study are available from the corresponding author upon reasonable request.
